# Spatial Heterogeneity in Cytoskeletal Mechanics Response to TGF-β1 and Hypoxia Mediates Partial Epithelial-to-Meshenchymal Transition in Epithelial Ovarian Cancer Cells

**DOI:** 10.3390/cancers15123186

**Published:** 2023-06-14

**Authors:** Deepraj Ghosh, Jeffrey Hsu, Kylen Soriano, Carolina Mejia Peña, Amy H. Lee, Don S. Dizon, Michelle R. Dawson

**Affiliations:** 1 Department of Molecular Biology, Cell Biology and Biochemistry, Brown University, Providence, RI 02912, USA; deepraj_ghosh@brown.edu (D.G.); carolina_mejia_pena@brown.edu (C.M.P.); 2 Center for Biomedical Engineering, Brown University, Providence, RI 02912, USA; amy_lee1@alumni.brown.edu; 3 Lifespan Cancer Institute, Warren Alpert Medical School of Brown University, Providence, RI 02912, USA; don_dizon@brown.edu; 4 School of Engineering, Brown University, Providence, RI 02912, USA

**Keywords:** EMT, hypoxia, ovarian cancer, mechanobiology, intratumor heterogeneity, single cell analysis

## Abstract

**Simple Summary:**

One major challenge to understanding and targeting deadly epithelial ovarian cancer is the heterogeneous nature of the disease, including intrinsic physical and genetic differences in cells and differences in the spatial microenvironmental cues in the tumor. In this study, we show that microenvironmental cues that mimic tumor promoting aspects of the in vivo tumor niche can alter the pattern of clustered epithelial cancer cells and induce the formation of invasive subpopulations. We identified the invasive subpopulations based on analysis of single cell physical characteristics and underlying structural protein organization. Our analysis highlights that peripheral cancer cells in a cluster display increased invasiveness in response to the external cues.

**Abstract:**

Metastatic progression of epithelial ovarian cancer (EOC) involves the partial epithelial-to-mesenchymal transition (EMT) of cancer cells in the primary tumor and dissemination into peritoneal fluid. In part to the high degree of heterogeneity in EOC cells, the identification of EMT in highly epithelial cells in response to differences in matrix mechanics, growth factor signaling, and tissue hypoxia is very difficult. We analyzed different degrees of EMT by tracking changes in cell and nuclear morphology, along with the organization of cytoskeletal proteins. In our analysis, we see a small percentage of individual cells that show dramatic response to TGF-β1 and hypoxia treatment. We demonstrate that EOC cells are spatially aware of their surroundings, with a subpopulation of EOC cells at the periphery of a cell cluster in 2D environments exhibited a greater degree of EMT. These peripheral cancer cells underwent partial EMT, displaying a hybrid of mesenchymal and epithelial characteristics, which often included less cortical actin and more perinuclear cytokeratin expression. Collectively, these data show that tumor-promoting microenvironment conditions can mediate invasive cell behavior in a spatially regulated context in a small subpopulation of highly epithelial clustered cancer cells that maintain epithelial characteristics while also acquiring some mesenchymal traits through partial EMT.

## 1. Introduction

Serous epithelial ovarian cancer (EOC) is the most lethal gynecological malignancy often diagnosed at late stages after the cancer has metastasized [1]. One major challenge to understanding and targeting ovarian cancer is the heterogeneous nature of the disease, which is the variance in morphology and gene expression across cancer cells and which makes it difficult for chemotherapeutic agents to target and kill all cancer cells [2,3]. Intratumor heterogeneity is also significantly higher in primary tumors compared to metastatic tumors since metastatic tumors develop from a subpopulation of cancer cells that are capable of metastasis [4]. The heterogeneity in primary tumor cells stems from intrinsic differences in cancer cells, along with host-selection pressures from the surrounding environment. Cancer cells also exhibit heterogeneity in vitro, which is influenced by chemical and physical aspects of the environment, including growth factor signaling and matrix mechanics [4,5]. In vitro studies of ovarian cancer have primarily focused on adherent cells grown on rigid plastic surfaces; however, mechanical forces transferred to cells from their surrounding environment can alter cancer cell behavior and influence cancer progression at all stages [6,7,8]. Moreover, the mechanical environment predominantly determines phenotypic and biophysical properties of a cell through mechanically dependent transcriptional reprogramming and cytoskeletal remodeling processes [9,10].

Studies in cancer mechanobiology have shown that cancer cells respond in a type-specific way to changes in matrix mechanics, with 70% of cancer cells displaying more aggressive behaviors on stiff vs. soft matrices and the remaining cell lines displaying matrix-independent behaviors [11]. In a previous study, we identified invasive cancer cell lines originating from different locations which undergo inverse responses to altered matrix rigidity: MDA-MB-231 breast cancer cells that prefer stiff matrices and SKOV-3 ovarian cancer cells that prefer soft matrices as characterized by increases in tumor cell growth, chemoresistance, and migration on the preferred mechanical environment [12,13]. Interestingly, these studies also showed that differential responses could arise within the same cancer type. For instance, more metastatic SKOV-3 ovarian cancer cells were shown to have greater mechanical tropism (increased traction forces and migration on softer substrates) compared to less invasive OVCAR3 ovarian cancer cells, which displayed similar traction force and migration rates on soft and hard substrates [12]. In addition, a small subpopulation of SKOV-3 cells displayed a much larger response to soft vs. hard substrates than other cells in the same population; these super responders highlight the heterogeneity seen in cancer cells. Here, we sought to determine if subpopulations of the less invasive ovarian cancer cell line (OVCAR3) were capable of responding to mechanical and chemical perturbations in the surrounding environment. To our knowledge, no comprehensive biophysical studies have been performed on the less invasive ovarian cancer cell response to both mechanical and chemical stimuli. Therefore, we revisited this OVCAR3 mechanical response model to determine if a combination of mechanical and chemical stimuli would elicit a more pronounced response in the average or outlier cell phenotype. The more epithelial phenotype of OVCAR3 cells is a more realistic model of the majority of cancer cells that likely exist in EOC tumors.

The epithelial-to-mesenchymal transition (EMT), whereby less motile regularly shaped polygonal epithelial cells transition into elongated invasive mesenchymal cells, is critical for EOC metastasis [14]. Markedly, changes in EMT processes are typically associated with lower expression of epithelial markers (e-cadherin, occludin, keratin) and higher expression of mesenchymal markers (n-cadherin, vimentin, fibronectin) [15]. Cytoskeletal remodeling is an essential part of EMT, which produces biophysical changes in cancer cells [16,17,18]. Epithelial cells lose their apical-basal polarity causing dissolution of cortical actin structure as the cells gain the more elongated mesenchymal morphology with front to rear polarity. The corresponding decrease in polymerized actin leads to softening of the cell cytoplasm and nucleus, making it easier for metastatic cells to navigate through dense extracellular environments [19,20]. Keratins that give epithelial cells their characteristic cuboidal shape are downregulated during EMT, which allows for cells to develop a more diverse and fluid-like structure [21,22]. The reduction in cytokeratin is important in cancer cell progression to mesenchymal phenotypes and increased migration capacities because it reduces the actin-cytokeratin interactions in the periphery of cells, thereby allowing more morphological alterations to occur [21,23]. In addition, some epithelial cancer cells lose only some epithelial characteristics, demonstrating both mesenchymal and epithelial features of a hybrid phenotype. This phenomenon is denoted as partial EMT and is prevalent in highly epithelial cancers such as EOC [24,25,26,27].

Transforming growth factor-β (TGF-β), a pleiotropic cytokine, is a potent inducer of EMT [28,29]. Elevated TGF-β levels are found in the serum and ascites of more aggressive EOCs [30]. Local levels of TGF-β in the tumor microenvironment can be maintained through autocrine signaling of cancer cells or paracrine signaling between cancer cells and stromal fibroblasts to promote optimal levels of TGF-β for tumor progression [31]. TGF- β can lead the conversion into mesenchymal states via SMAD signaling and nuclear translocation of EMT transcription factors, Snail-1 and Snail-2 [28]. EMT can also arise from SMAD-independent TGF- β signaling, which can activate the PI3K-AKT pathway to stimulate cell growth and Rho-GTPase signaling to promote actin stress fiber, lamellipodia, and filopodia formation [32]. Similarly, during matrix-induced EMT signaling, mechanosensitive pathways, such as myosin contractility and Rho-ROCK signaling pathways, are activated to maintain cytoskeletal tension and induce cytoskeletal remodeling [33]. Oxidative stress and hypoxia in the tumor niche are among other powerful drivers of EMT-mediated invasive cancer cell behavior [34,35,36,37]. There is also crosstalk between the TGF-β signaling and hypoxia induced EMT transcription factor activation [38]. Moreover, hypoxia induced the expression of immunosuppressive molecules in tumor cells, which could potentiate EMT by enhancing TGF-β signaling [39].

We hypothesized that chemical and mechanical stimuli would induce a more invasive cancer cell phenotype in a spatially regulated subpopulation of OVCAR3 cells that lie within these less invasive cell clusters. In order to investigate this hypothesis, we cultured OVCAR3 cells on soft and hard matrices with additional stimuli, including TGF-β and hypoxia treatment, to further enhance their invasive characteristics, as previous studies indicated that OVCAR3 cells remained unperturbed under mechanical stimulus alone. We then quantified high-magnification (40x) images for nuclear and cellular morphologies, followed by intensity analysis of spatial distribution of cytoskeletal proteins. These results showed that OVCAR3 cells developed a more elongated nuclear morphology and greater perinuclear expression of phosphorylated Myosin (p-Myo) when cultured in hypoxic conditions on soft substrates with TGF-β treatment (mechanical and chemical stimuli condition). However, the relatively high coefficient of the variations in cell and nuclear morphologies and insignificant changes in the average cytoskeletal protein expression among all conditions suggested that a cellular subpopulation was driving the significant changes in cytoskeletal and nuclear structure. Thus, we performed spatial analysis of epithelial cell clusters focusing on central and peripheral cell populations. We show that a greater percentage of peripheral cells were elongated under mechanical and chemical stimuli. Thereafter, we used specific cytoskeletal inhibitors to corroborate the chemically and mechanically dependent cytoskeletal changes in OVCAR3 cells. Collectively, these data show that tumor-promoting microenvironment conditions can mediate invasive cell behavior in a spatially regulated context in a small subpopulation of highly epithelial clustered cancer cells.

## 2. Materials and Methods

### 2.1. Materials

Cell culture reagents, including RPMI 1640 medium and penicillin-streptomycin, were purchased from Corning Incorporated (Tewksbury, MA, USA). Fetal bovine serum (FBS) was purchased Bio-Techne Sales Corporation (Minneapolis, MN, USA). Rhodamine-conjugated Phalloidin was obtained from Invitrogen (Waltham, MA, USA). Purified anti-Cytokeratin (pan reactive) antibody was obtained from Biolegend (Santa Clara, CA, USA). All other primary and secondary antibodies used in this study were purchased from Cell Signaling Technology (Danvers, MA, USA). Hoechst 33342 was purchased from Invitrogen. Small molecular inhibitors were purchased from Cayman Chemical Company (Ann Arbor, MI, USA). All other chemicals and materials were purchased from Fisher Scientific unless specified otherwise.

### 2.2. OVCAR3 Cell Culture

Epithelial Ovarian Cancer (EOC) cell line OVCAR3 (ATCC, Manassas, VA, USA) was cultured in RPMI 1640 media supplemented with 10% Fetal Bovine Serum (FBS) and 1% penicillin-streptomycin. Cells were maintained at 5% CO_2_ and 37 °C.

### 2.3. TGF-β stock preparation

Recombinant human protein TGF-β1 was purchased from Biolegend (Santa Clara, CA, USA), and stocks were made in PBS containing 0.2% bovine serum albumin (BSA). Stocks were aliquoted and stored in −80 °C until use.

### 2.4. Polyacrylamide Substrate Synthesis (Soft and Hard)

Two-dimensional tunable collagen-coated polyacrylamide (PA) substrates of varying stiffnesses were synthesized according to published methods [40]. Briefly, glass coverslips were initially cleaned with 0.1M sodium hydroxide and then functionalized with single consecutive treatments of 1% (*v*/*v*) 3-aminopropyltrimethoxysilane for 10 min and 0.5% glutaraldehyde (*v*/*v*) for 30 min. A final solution of 10% acrylamide (*w*/*v*) with 0.03% or 0.3% bis-acrylamide (*w*/*v*) was polymerized on activated glass coverslips to generate soft (2.6 kPa) and hard (36.2 kPa) substrates, respectively (Table A1). Ammonium persulfate and Tetramethlethylenediamine (TEMED) catalyzed the polymerization of acrylamide mixtures on glass coverslips. Soft and hard substrates were then crosslinked with 0.1 mg/mL type I collagen solution using Sulfo-SANPAH (2 mg/mL) prior to cell culture.

### 2.5. TGF-β and Hypoxia Treatment

OVCAR3 cells were serum starved overnight prior to a 24 h treatment with 50 ng/mL of TGF-β. Cells were collected and harvested for further downstream experiments. Hypoxia—OVCAR3 cells were maintained at reduced oxygen conditions of 2.5% O_2_ for 24 h. Combination Treatment—OVCAR3 cells were serum starved overnight under normoxic conditions. Cells were then treated with 50 ng/mL of TGF-β under hypoxic conditions for 24 h.

### 2.6. Cytoskeletal Inhibitors

Small molecule inhibitors ML7 (targeting myosin light chain kinase), H1152 (targeting ROCK), and CCG1423 (targeting MRTF) were dissolved in DMSO and stored in −20 °C. OVCAR3 cells were serum starved overnight prior to a 24 h treatment with DMSO (vehicle control), ML7 (10 µM), H1152 (1 µM), or CCG1423 (10 µM).

### 2.7. Immunofluorescence staining

OVCAR3 cells cultured on polyacrylamide substrates were stained after treatment period to analyze nuclear and cytoskeletal properties according to previously published protocol [12]. Briefly, cells were fixed with 4% paraformaldehyde, permeabilized with 0.5% Triton X-100, and blocked with 5% goat serum. Cells were then stained with a combination of primary antibodies (anti-Phospho-Myosin IIA (1:200) and anti-Cytokeratin (1:100)) in 2% BSA overnight at 4C. Next day, cells were washed and stained with Rhodamine Phalloidin (1:200), AF-488-conjugated anti-rabbit IgG (1:500), AF-647-conjugated anti-mouse IgG (1:500), and Hoechst 33342 (1:5000). Coverslips were mounted using Vectashield and imaged with a Nikon Eclipse Ti inverted fluorescence microscope at 40x magnification.

### 2.8. Cell Cluster Analysis

Cell cluster analysis was performed manually for individual images in FIJI (NIH). A “cluster” was defined as a collection of adjoined cells that exhibited overlapping actin cytoskeletal structures at cell–cell boundaries. Furthermore, the centroid of two nuclei were required to be less than twice the distance of the nuclear diameter away for all the cells to be considered in a single cluster. Stained nuclei were used to assess the number of single cells in each cluster. Single peripheral cells were distinguished as a cell that was located on or near the outer border of cell clusters and that had less than 25% of its cell boundary in contact with another cell. These cells showed distinct polarized actin structures. Additionally, the centroids of these nuclei were greater than 2x the distance of the nuclear diameter away from any other nuclear centroid.

### 2.9. Cluster Cell and Nuclear Shape Factor Analysis

Cell and nuclear morphologies for cluster analysis were performed using a FIJI shape parameter tool. Cell shape factor (CSF) and nuclear shape factors (NSF) values of 1 represented a circle, and values of 0 represented a line.

### 2.10. CellProfiler and MATLAB analysis

**Figure A1:**
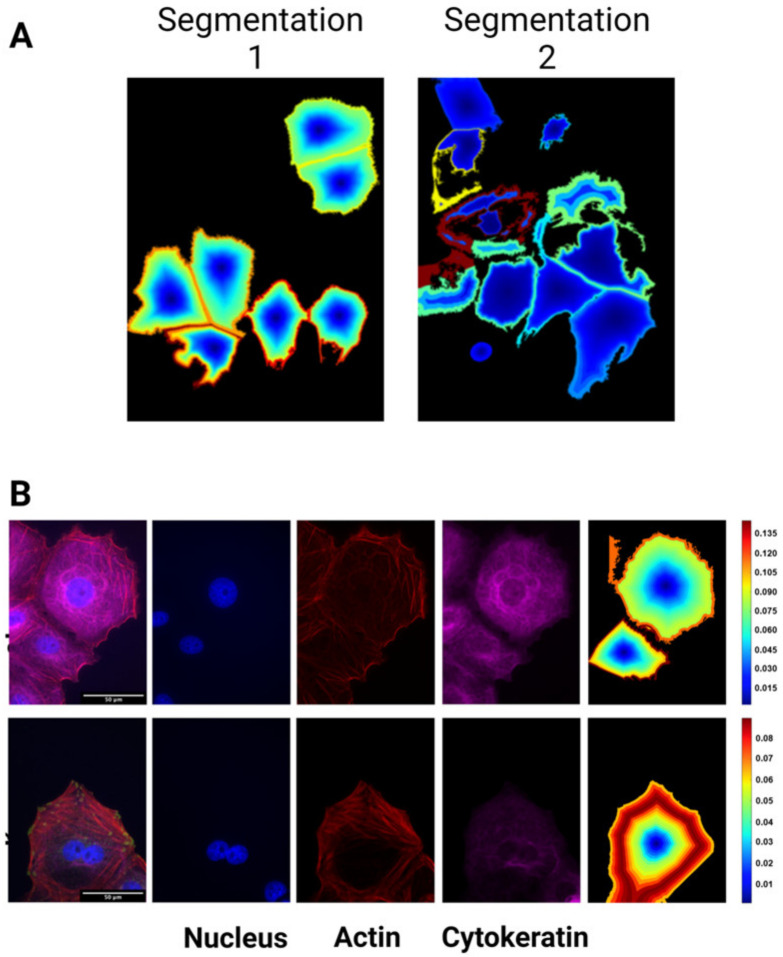
CellProfiler pipeline output examples showing segmentation of clustered cell boundaries (**A**) and heatmap of radial protein distribution in OVCAR3 cells (**B**).

We used CellProfiler (Broad Institute, MIT) to analyze immunofluorescent images of OVCAR3 cells stained with F-actin, p-Myo, and Cytokeratin antibodies (Figure A1). With this platform, we generated high content single cell data containing the morphological properties (area, size, shape factors) as well as cytoskeletal organization (pattern of cytoskeletal proteins) for each cell. Mean intensity (a.u.) for each protein image for each cell was calculated using default CellProfiler settings that normalized the raw image intensity from 0 to 1 by dividing all pixels in the image by the maximum possible intensity value. We post-processed the single cell data using custom written MATLAB code to perform further analysis on the generated datasets. For quantile analysis of any parameter (e.g., nuclear or cell shape factor), we used MATLAB to find the cutoff value for each parameter and sorted the high content data passing the defined threshold.

### 2.11. Statistics

Statistical analysis was performed using GraphPad Prism. Experimental data from three independent biological replicates were pooled for single cell heterogeneity analyses. We first calculated the average and the coefficient of variation (CV) in Prism. We used two-way ANNOVA (for >2 groups) with Tukey’s multiple comparison test to determine statistical significance. Statistical significances were indicated as (*) for *p* ≤ 0.05, (**) for *p* ≤ 0.01, (***) for *p* ≤ 0.001, and (****) for *p* ≤ 0.0001 for the graphs. Statistical significance for the tabulated values was indicated as an underline for all *p*-values less than 0.05.

## 3. Results

### 3.1. OVCAR3 Cells Exhibit Altered Cellular and Nuclear Morphologies under Mechanical and Chemical Stimuli

**Figure 1:**
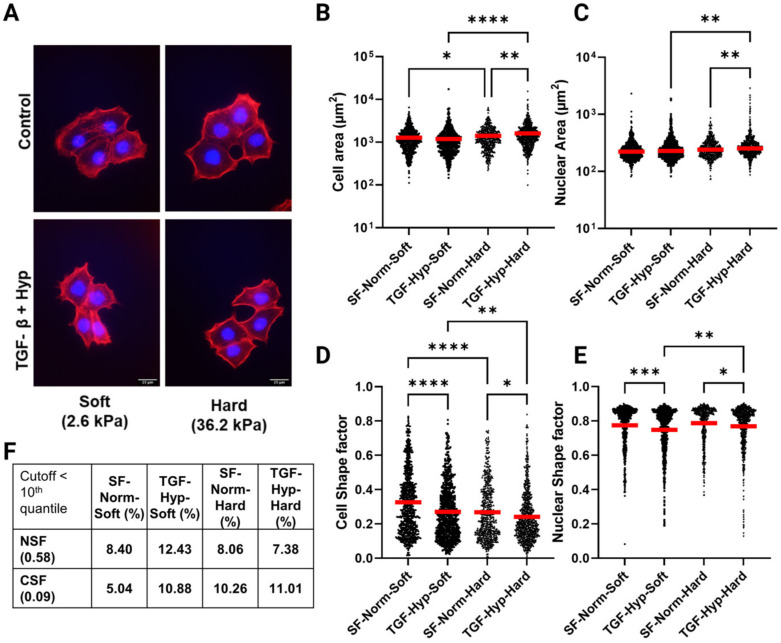
Single cell morphological analysis of OVCAR3 cells. High grade ovarian cancer cell line OVCAR3 was cultured on collagen coated polyacrylamide substrates of varying stiffness (Soft ~2.6 kPa and Hard ~36.2 kPa) in Normoxia and Hypoxia (~2.5% O_2_) and was treated with transforming growth factor-β1 (TGF-β1) at a concentration of 50 ng/mL for 24 h. Cells were then fixed and stained to quantify the cell and nuclear shape. (**A**) OVCAR3 cells stained with Phalloidin (red) and DAPI (blue) were imaged on Nikon inverted Ti-E microscope at 40x magnification (Scale bar = 25 µM). (**B**–**E**) Fluorescent images were analyzed using CellProfiler to quantify cell and nuclear shape properties. We quantified more than 500 cells spanning 3 independent experiments for each condition (N = 3). (**B**,**C**) Both cell and nuclear areas remained unchanged after treatment on soft substrates. In contrast, both parameters were significantly upregulated on Hard substrates after treatment. (**D**,**E**) Cell and nuclear shape factors, measured to quantify elongation, were significantly downregulated on both substrates after treatment. On both substrates, an elongated subpopulation emerged after treatment. (**F**) Percentages of cells with NSF or CSF values in the 10th quantile, representing the top 10% most elongated cells. Red horizontal lines in the graphs indicate the mean value of the population. Statistical significances are indicated as (*) for *p* ≤ 0.05, (**) for *p* ≤ 0.01, (***) for *p* ≤ 0.001, and (****) for *p* ≤ 0.0001.

Morphological alterations are one of the most phenotypically representative hallmarks of cytoskeletal reorganization during cancer cell EMT. In order to probe nuclear and cellular morphologies of OVCAR3 cells under the influence of EMT-inducing chemical and mechanical environments, cells were fluorescently stained with DAPI for nuclei and phalloidin for F-actin and imaged at high magnification (40x) on varying substrate rigidities (soft, hard, TCP) and chemical cues (TGF-β1, Hypoxia) (Figure 1A). Soft and hard polyacrylamide–collagen matrices were made to physiologically represent Young’s moduli of adipose tissue (2.6 kPa) and tumor tissue (36.2 kPa), respectively [12]. Cells were then batch-processed with CellProfiler using an optimized pipeline for morphological quantification of cell area (Figure 1B), nuclear area (Figure 1C), cell shape factor (Figure 1D), and nuclear shape factor (Figure 1E). The shape factor (SF) was used to characterize cell and nuclear elongation, with a shape factor of 1 indicating a perfect circle and 0 indicating a straight line.

Consistent with previous studies, the OVCAR3 cells exhibited minimal response to mechanical perturbation alone, as evidenced by the limited changes in nuclear and cellular area (Figure 1B,C). We only found modest increases in cell and nuclear area on hard substrates post treatment (Figure 1B,C). Interestingly, shape factor quantification of over 500 OVCAR3 cells and nuclei under TGF-β1, and hypoxia treatment on soft substrates showed a significant reduction in both cell (CSF) and nuclear shape factor (NSF) (Figure 1D,E), suggesting that synergistic chemical and mechanical cues could be sufficient to induce EMT-related morphological changes in this less invasive and more epithelial ovarian cancer cell line. We also found a more unique subpopulation of OVCAR3 cells with elongated nuclei (NSF < 0.5) in the TGF-β1 and hypoxia treatment group. However, the cell shape factor (CSF) measurements for the OVCAR3 cells on substrates with TGF-β1 and hypoxia treatment did not seem to follow the same observation.

Further analysis showed that this difference was likely due to the more heterogeneous nature of cell vs. nuclear morphologies, as evidenced by the larger coefficient of variation for cell-morphology-related parameters compared to those of nuclear morphology (Table A3). This was consistent with previous observations because cell deformation has been shown to be biologically easier to occur than nuclear deformation since the nucleus is highly compact and dense relative to the overall cellular architecture [41,42,43]. Hence, we suspected that there was a small subpopulation of EMT-induced cells with elongated morphologies and divergent cytoskeletal alterations that were overshadowed in the global analysis of cells treated with TGF-β1 and hypoxia. To test this hypothesis, we looked at the percentage of cells for each condition with NSF < 0.58 and CSF < 0.09; these cutoffs were based on NSF and CSF values for the 10th quantile, which represented the top 10% most elongated cells (Figure 1F, Table A3). This data showed the largest percentage of elongated nuclei in TGF-β1- and hypoxia-treated cells on soft substrates and increased percentages of elongated cells in treated and control groups on hard substrates (Figure 1F). Both elongated cell and nuclei subpopulation percentage increased more profoundly on soft substrates post-treatment.

**Figure A2:**
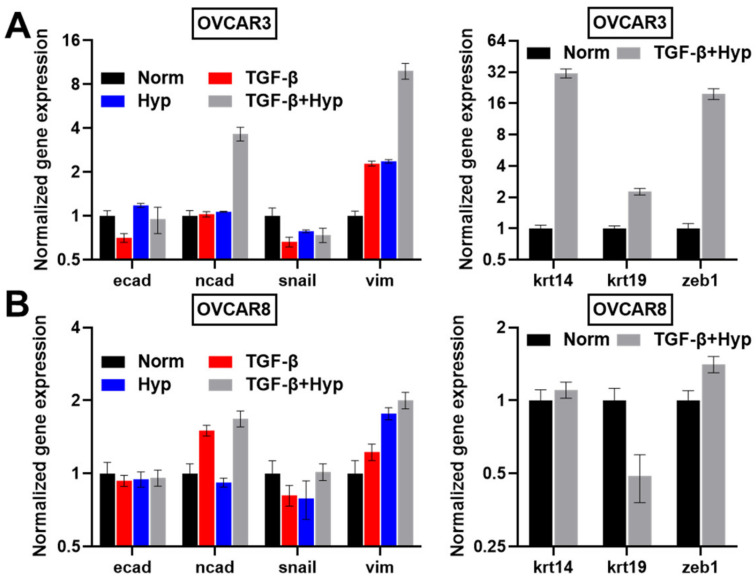
Gene Expression of EMT Markers in Ovarian Cancer Cells. Graph shows qRT-PCR data for (**A**) OVCAR3 and (**B**) OVCAR8 cells. EMT markers across four treatment conditions: (1) Control, (2) TGF-β, (3) Hypoxia, (4) TGF-β plus Hypoxia are shown on the left, including epithelial marker E-cadherin and mesenchymal markers N-cadherin, Snail, Vimentin. qRT-PCR experiments to assess the effects on cytokeratin and ZEB1 for the control and hypoxia with TGF-β1 treatment are shown on the right. Results are reported as average ± standard error of the mean (n = 2).

Our initial studies of bulk gene expression analysis with qRT-PCR of OVCAR3 cells demonstrated inconsistent genetic changes, confirming a lack of significant response even after TGF-β1 and hypoxia treatment (Table A2, Figure A2). We found that the expression of both mesenchymal markers, including N-Cadherin (*ncad*), Vimentin (*vim*), and ZEB1 (*zeb1*), and epithelial markers, including keratin-14 (*krt14*) and keratin-19 (*krt19*), increased to different degrees with TGF-β and hypoxia treatment in OVCAR3 on tissue culture plastic (Figure A2).

### 3.2. Reduced Size of OVCAR3 Cell Clusters under Mechanical and Chemical Stimuli

**Figure 2:**
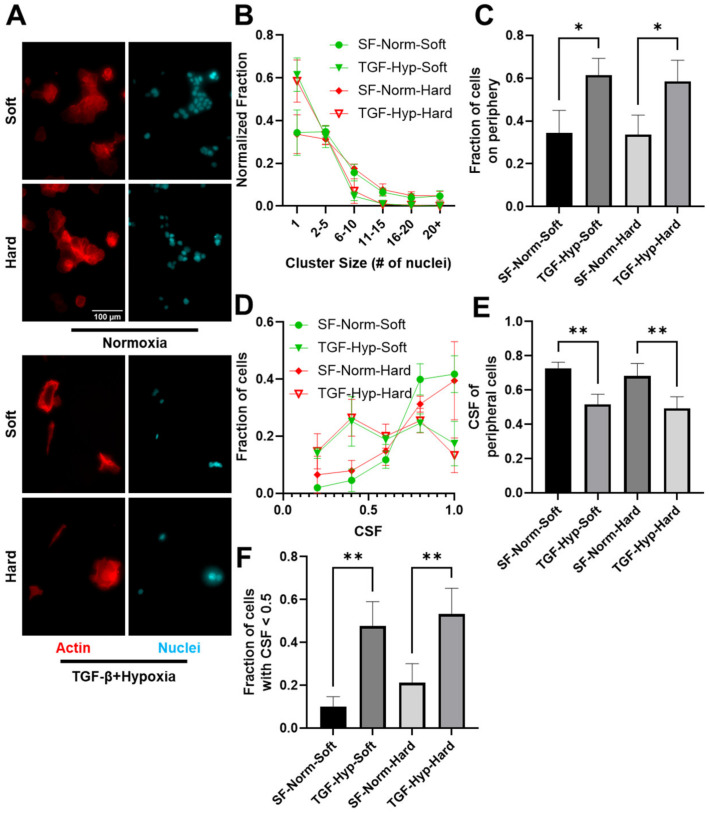
Cell Cluster Characterization on Soft and Hard Substrates. To analyze change in cluster size of OVCAR3 cells treated with TGF-β1 and hypoxia on soft and hard substrates, images were analyzed manually using Image J. (**A**) Cells were stained with Phalloidin (F-actin, red) and DAPI (nucleus, blue). (Scale bar = 100 um). (**B**) The number of cells in a cluster was measured by the number of nuclei identified in that given cluster. Quantification of single cells or clusters of cells ranging from very small (2–5 cells) to very large clusters (20+) for all the conditions revealed an increase in single cell subpopulations on both soft and Hard substrates. (**C**) Number of cells on the periphery of these clusters also increased for both stiffnesses. (**D**) Histograms show the count of cells for all CSF values for the OVCAR3 single periphery cells treated with TGF-β plus hypoxia plated on soft and hard substrates. (**E**) Graph shows mean CSF for the entire single periphery cell population in all three treatment conditions. (**F**) Graph shows the percentage of cells registering a CSF below 0.5 for periphery cells in all conditions. Results are reported as average ± standard error of the mean (N = 3). Statistical significances are indicated as (*) for *p* ≤ 0.05, (**) for *p* ≤ 0.01.

To further understand the effects of mechanical and chemical stimuli on cancer cell EMT, we next looked at the size of OVCAR3 cell clusters. Cells were either untreated or treated with both TGF-β1 and hypoxia and then plated on substrates (Figure 2A). Individual clusters were identified based on their overlapping actin structure and close nuclear proximity, with a distance less than 2x the nuclear diameter considered to be part of the same cluster. Cells were stained with DAPI and Phalloidin to assess the number of cells per cluster (Figure 2A). Heterogeneity analysis of all cells in the population yielded two distinct subpopulations: (1) cells packed together in a cluster (denoted as cluster cells), and (2) cells at the periphery of these clusters (denoted as periphery cells). We also quantified single periphery cells, which were defined as cells with no overlap in actin staining and nuclei more than 2x the nuclear diameter away from other nuclei. There was an increase in smaller clusters, including single periphery cells and clusters of two–five cells, for TGF-β- and hypoxia-treated conditions on both soft and hard substrates, whereas the serum-free control populations had an increased fraction of larger clusters, with six plus cells (Figure 2B). In fact, TGF-β and hypoxia treatment resulted in an increased fraction of single periphery cells (Figure 2C). In many of the periphery cells we observed an elongated actin filament cytoskeleton, whereas central cluster cells maintained a more cobblestone appearance. The fraction of periphery cells with CSF less than 0.5 significantly increased for TGF-β and hypoxia treatment groups (Figure 2D), which may have been indicative of EMT in treated cells. This increased fraction of elongated cells resulted in reduced mean CSF for treated vs. control cells (Figure 2E,F). This indicated that the TGF-β- and hypoxia-treated periphery cells were more elongated than untreated periphery cells on both the soft and hard substrates. This population of elongated periphery cells would be lost in bulk analysis. This demonstrated a key finding in the understanding of the EMT.

### 3.3. Heterogeneity in Cytoskeletal Protein Expression

**Figure 3:**
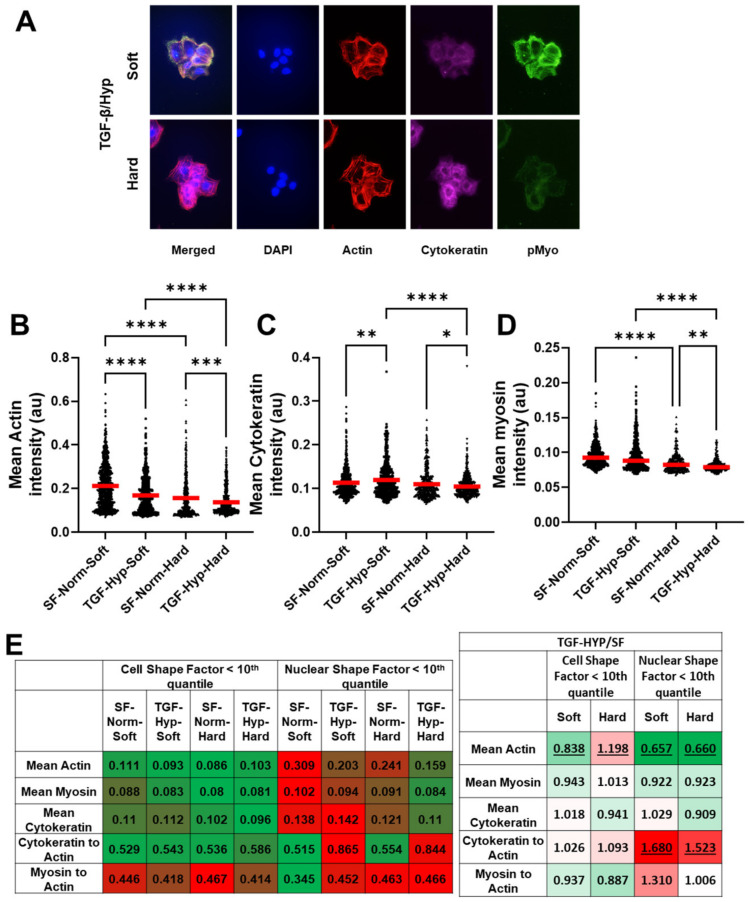
Cytoskeletal reorganization of OVCAR3 cells. (**A**–**D**) To visualize cytoskeletal organization, OVCAR3 cells were stained for p-Myosin (green), Actin (red), and Cytokeratin (Cyan). Mean intensity of the cells was quantified using CellProfiler. (**B**) Mean Actin intensity was significantly reduced on both soft and hard substrates after treatment with a combination of hypoxia and TGF-β1. (**C**) Upon treatment, cytokeratin intensity was increased on soft substrates but decreased on hard substrates. (**D**) Myosin expression was significantly reduced on hard substrates but not on soft substrates. (**E**) Heat maps of the mean intensities of actin, cytokeratin, and p-Myo staining for cells with CSF or NSF values in the 10th quantile (which represent the cells with the top 10% most elongated cell and nuclear structure) and hypoxia- and TGF-β1-treated groups normalized to serum-free controls. Green represents the lowest value and red represents the highest value in each row of the tables. These data show elongated cell shape correlates with decreased intensities of all three cytoskeletal proteins, whereas elongated nuclear morphology correlates with increased intensities for all groups except hypoxia- and TGF-β1-treated cells on hard substrates. Furthermore, TGF-β1 and hypoxia treatment resulted in dramatic decrease in actin expression and increase in cytokeratin-to-actin ratio in cells with elongated nuclear morphology, indicating this phenotype was likely associated with partial EMT where both epithelial and mesenchymal traits are expressed. Statistical significances are indicated as (*) for *p* ≤ 0.05, (**) for *p* ≤ 0.01, (***) for *p* ≤ 0.001, and (****) for *p* ≤ 0.0001. Statistical significance for the tabulated values in figure E (panel 2) are indicated as underlines for all *p*-values less than 0.05.

**Figure A3:**
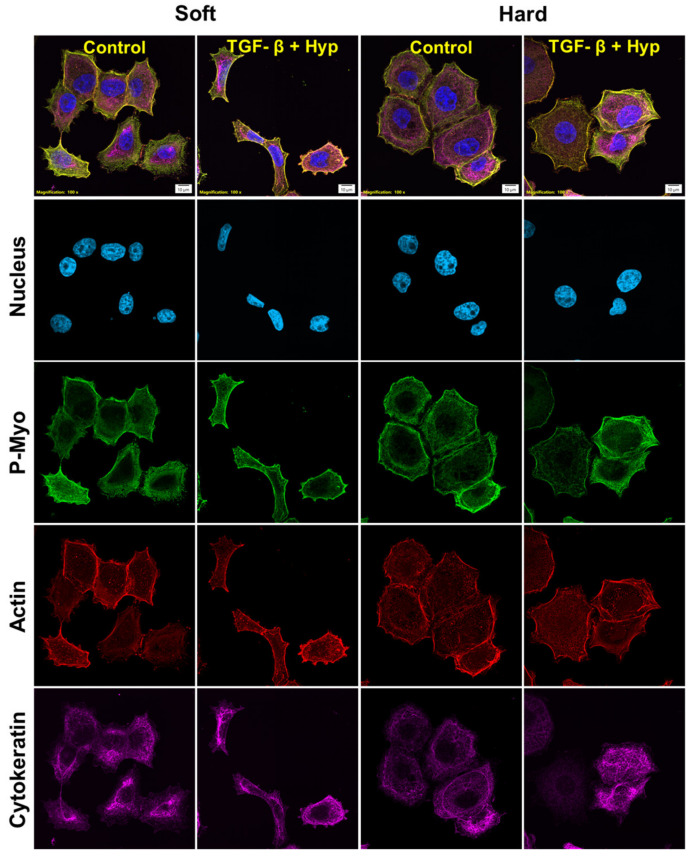
High magnification confocal images of OVCAR3 cells cultured on polyacrylamide substrates (soft and hard) and treated with a combination of TGF-β1 (50 ng/mL) and hypoxia (2.5% O_2_) for 24 h. Cells were stained for nucleus (blue), phospho-myosin (green), actin (red), and cytokeratin (magenta) and then imaged on a confocal microscope at 100x magnification (Scale bar 10 um).

**Figure A4:**
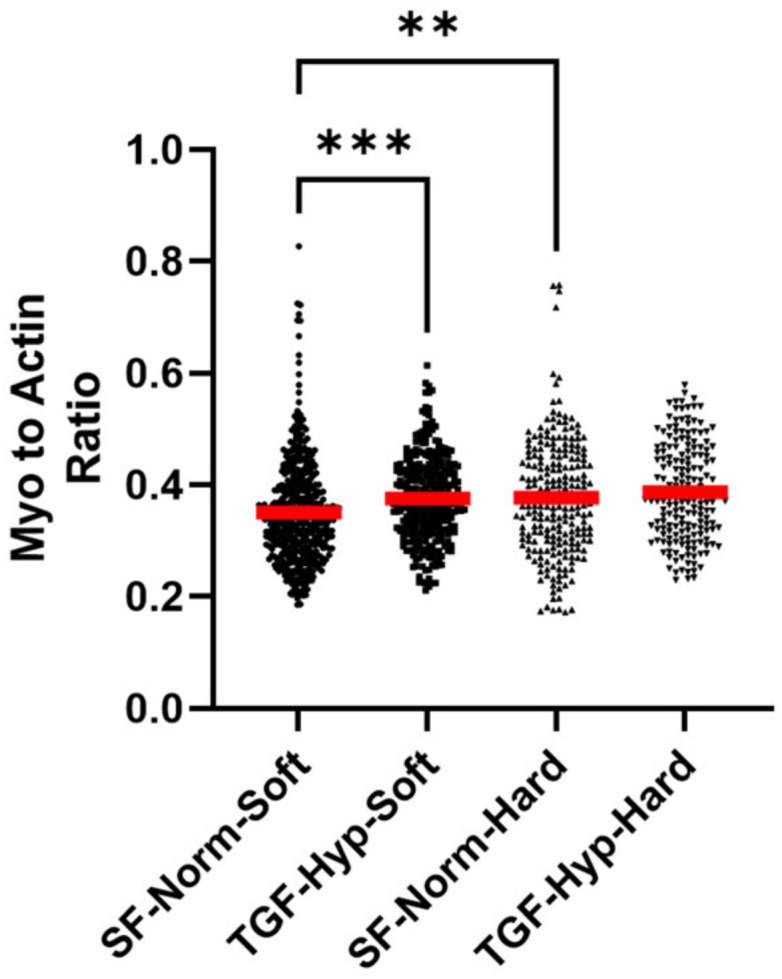
Phosphorylated myosin-to-actin ratio for OVCAR3 cells treated with a combination of TGF-β1 and hypoxia on soft and hard polyacrylamide substrates. Red horizontal lines in the graphs indicate the mean value of the population. Statistical significances are indicated as (**) for *p* ≤ 0.01, and (***) for *p* ≤ 0.001.

We further investigated the effects of mechanical and chemical stimuli on EMT by looking at the differences in cytoskeletal protein expression for F-actin (red), pan-cytokeratin (cyan), and p-Myo (green) in control and treated single cell populations cultured on soft and stiff substrates (Figure 3A and Figure A3). Based on the mean fluorescence intensities, we observed a decrease in F-actin and pan-cytokeratin expression, with a corresponding reduction in the expression of p-Myo in OVCAR3 cells on hard substrates treated with TGF-β1 and hypoxia (Figure 3B–D). On soft substrates, only F-actin expression was reduced significantly post-24 h treatment period, whereas cytokeratin expression increased. Cytokeratin is a hallmark of epithelial phenotype, and reduced cytokeratin expression has been implicated in cancer cell EMT. Moreover, cuboidal epithelial cells lose their apical-basal polarity during EMT, which causes the dissolution of the cortical actin structure, as cells gain a more elongated mesenchymal structure. Furthermore, the p-Myo to F-actin ratio remained similar for most conditions with a modest increase on soft substrates post treatment (Figure A4). Next, we looked at the mean fluorescent intensities of F-actin, cytokeratin, and p-Myo staining for the 10th quantile of nuclear and cell shape factors, which correspond with top 10% most elongated nuclei and cells (Figure 3E). The mean fluorescence intensities of F-actin were lower than average in the most elongated cells (10th quantile for CSF); the expression of all three proteins were higher than average in cells with the most elongated nuclei (10th quantile for NSF), indicating these cell populations were likely different. In both elongated populations (10th quantiles for NSF and CSF), TGF-β and hypoxia treatment resulted in reduced mean intensities for F-actin on soft substrates. On stiffer substrates, the mean actin intensity increased in elongated cells but decreased in cells with elongated nuclei. Furthermore, cells with elongated nuclei had similar reductions in p-Myo and cytokeratin expressions on both substrates and a significant increase in the cytokeratin-to-actin ratios. The increased cytokeratin-to-actin ratio likely indicated that cells lost some of their cortical actin but maintained epithelial levels of cytokeratin expression, which was characteristic of a partial EMT response, where cells displayed both epithelial and mesenchymal cell features.

### 3.4. Radial Distribution in Cytoskeletal Protein Expression

**Figure 4:**
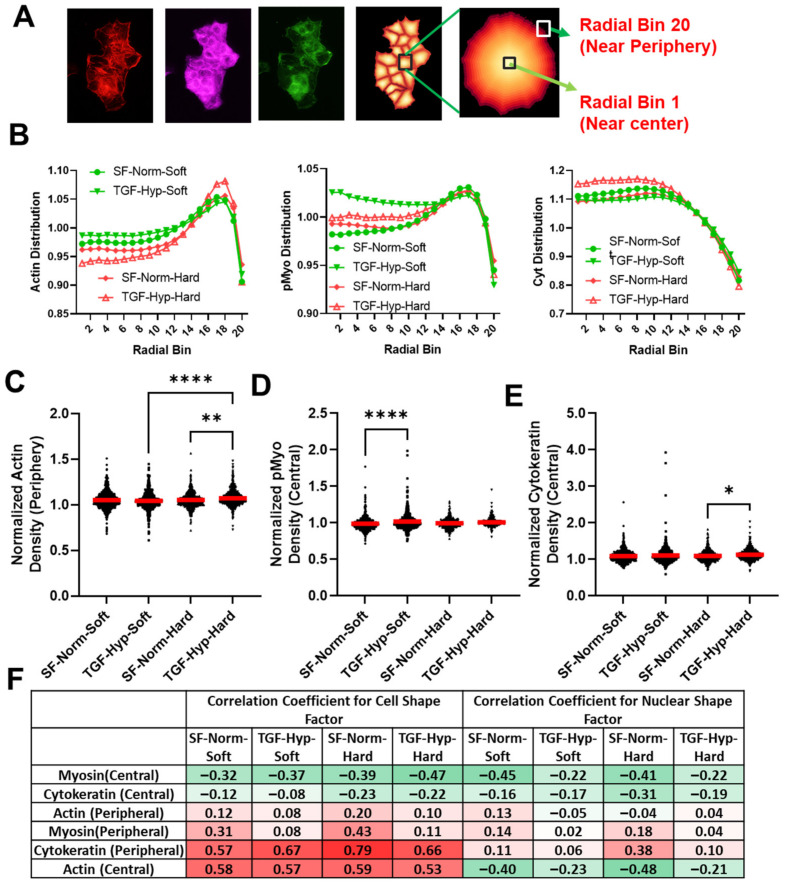
Intracellular distribution of cytoskeletal proteins in OVCAR3 cells. (**A**) To analyze intracellular organization of intracellular actomyosin and cytokeratin network, we used CellProfiler to quantify the normalized fraction of protein expression in 20 continuous regions (bins) spanning between cell center (bin 1) and cell periphery (bin 20). (**B**) Distribution of actin showed higher concentration of proteins near the periphery of the cells; whereas p-Myo expression was more evenly distributed, and cytokeratin expression was highest near the central region of the cells. Treatment with the combination of TGF-β1 and hypoxia resulted in a change in distribution of actin near the periphery and cytokeratin near the center for hard substrates. This also resulted in increased p-Myo distribution near the center for soft substrates. (**C**) The normalized intensity of actin near the periphery (bin 18) were significantly increased on hard substrates after treatment. (**D**,**E**) The normalized intensity of p-Myo near the center (bin 3) was increased significantly on soft substrates, whereas the intensity of cytokeratin near periphery was increased on hard substrates. (**F**) To determine if changes in cell and nuclear shape corresponded with the expression of cytoskeletal proteins, we show a heat map of correlation coefficients for the distribution of protein expression at the center (bin 3) or periphery (bin 18) relative to cell or nuclear form factor. Green represents the lowest value (−1) and red represents the highest value (+1) in the heatmap of the correlation coefficients. These data show central p-Myo resulted in the most elongated cell and nuclear phenotypes, whereas increased peripheral cytokeratin and central F-actin were associated with rounder cell morphology. Interestingly, central F-actin also modestly corelated with increased nuclear elongation. Red horizontal lines in the graphs indicate the mean value of the population. Statistical significances are indicated as (*) for *p* ≤ 0.05, (**) for *p* ≤ 0.01, and (****) for *p* ≤ 0.0001.

A radial distribution protocol in CellProfiler was utilized to quantify the physical location of cytoskeletal proteins, including F-actin (red), cytokeratin (cyan), and p-Myo (green) from the center to periphery of the cells based on fluorescence intensities. The radial distribution analyzed the fluorescence intensity from the perinuclear region of the cell (bin 1) to the cell cortex (bin 20), normalized by the cell’s mean fluorescence intensity for each fluorophore (Figure 4A). Radial distribution graphs showed the fraction of F-actin, p-Myo, and cytokeratin that was expressed in the cell from bin 1 to bin 20 (Figure 4B). The normalized intensities of F- actin and p-Myo were highest at the cell cortex, whereas the intensity of cytokeratin was highest in the perinuclear region of the cell. The increased fraction of polymerized actin and p-Myo at the periphery of cells likely supported their more dynamic role in maintaining the cell shape under cytoskeletal tension, which required actomyosin-generated contractile forces at sites with higher expression of p-Myo. Whereas the increased fraction of cytokeratin in the perinuclear region of the cell likely supported its more stable role in providing mechanical stability to the cell’s nucleus. Toward this end, we saw much larger changes in cell shape (Figure 1D) than in nuclear shape (Figure 1E) for all conditions, where the variation in cell shape was more likely governed by the organization of F-actin and nuclear shape changes that were more likely to be buffered by a more stable expression of cytokeratin.

However, the effects of mechanical and chemical stimuli on average radial distributions of the three cytoskeletal proteins were modest and somewhat inconsistent (Figure 4C–E). We found an increase in central p-Myo on soft substrates, whereas increased peripheral actin and central cytokeratin were observed on hard substrates after treatment. To determine if the intracellular protein distribution was correlated with more elongated or round morphologies, we determined the correlation coefficients between protein distribution and cell and nuclear shape factors. The correlation coefficient varies between negative (−1) and positive (+1) values of one. For example, an increased protein localization with a negative and positive correlation coefficient for a shape factor aa more elongated and rounder morphology, respectively (Figure 4F). Increased expression of central p-Myo was associated with more elongated cell and nuclear phenotypes, whereas peripheral p-Myo was associated with a slight increase in cell and nuclear shape factors, i.e., rounder cells (Figure 4F). Increased expression of central cytokeratin was moderately associated with elongated cell and nuclear shape profiles. Peripheral cytokeratin and central actin were both associated with rounder cell morphologies; however, nuclei remained round with peripheral cytokeratin and became more elongated with central actin. This finding was crucial since a decrease in cytokeratin expression in the periphery of cancer cells suggested less actin-cytokeratin interactions in the periphery and subsequent increase in cell–matrix interactions for potential cancer cell invasion. A decrease in cytokeratin expression at the cortex of a migrating cell may have also created more space for actin microfilaments to creep in and form actin-based protrusions, which were important during cancer cell invasion and metastasis. Furthermore, cells with increased expression of central p-Myo and F-actin were more likely to have elongated nuclei in response to the treatment with TGF-β1 and hypoxia, suggesting that cytoskeletal tension may have been important in this response.

### 3.5. Morphological Differences in Cells with High and Low Cytoskeletal Protein Expression

Next, we looked at cell and nuclear shape factors for cells with the highest (top 10%) and lowest (bottom 10%) levels of cytoskeletal protein expression (Table 1A). Low actin expression was correlated with a more elongated cell morphology with rounder nuclear structures, whereas high actin expression was correlated with more elongated nuclear structure and less elongated cell morphology. This trend was similar for myosin low and myosin high expression, although the myosin low population was initially rounder than the actin low and myosin high much rounder than actin high populations. The cytokeratin low expression also displayed a more elongated cell morphology and rounder nuclear structure; however, high cytokeratin expression was associated with elongated cells and nuclear structures. The ratio of cytokeratin to actin expression heavily affected cell morphology, with higher ratios of cytokeratin to actin associated with more elongated cell structures. The myosin-to-actin ratio also corresponded nicely with differences in cell and nuclear shape, with low values indicating more elongated nuclear structures and high values more rounded cell structures for all conditions.

To determine if differences in cytoskeletal structure were correlated with the cell’s ability to respond to chemical and mechanical stimuli, we determined the ratio of cell and nuclear shape factors for TGF-β1- and hypoxia-treated cells relative to serum-free control cultured on soft and hard substrates (Table 1B). The conditions highlighted in green demonstrated more elongated morphologies and those highlighted in red rounder morphologies, with those that are outlined being statistically significant differences relative to control. Overall, cell shape factor was much more dynamic than nuclear shape factor (which remained relatively constant), and we saw differences in cell morphology for most chemical and mechanical perturbations. Cells with high actin and myosin expression or low cytokeratin-to-actin ratio were more likely to elongate on the soft substrates; whereas both low and high cytokeratin cells were more likely to elongate on hard substrates. In addition, cells with low myosin expression were more likely to display rigidity independent behavior. This result corresponded well with our previous mechanical tropism study where we showed that breast cancer cells that preferred hard substrates had higher actomyosin contractility gene expression than ovarian cancer cells that preferred soft substrates. Here, we showed that there were subpopulations of cells with different mechanical responses to substrate stiffnesses even within a single cell type. Nuclear elongation was only seen in a few conditions, including the actin-lo cells on hard substrates, cells with high cytokeratin-to-actin ratios on hard substrates, and cells with high myosin-to-actin ratios on soft substrates. For cells grown on the hard substrates, cells with more elongated nuclear structures had rounder cell morphologies, whereas cells on soft substrates displayed more elongated nuclear and cell structures. In the case of actin-lo on the hard and the high myosin-to-actin ratio on the soft, the nuclear shape factor was decreased relative to the other conditions with the change in matrix stiffness; however, for the cytokeratin to actin high on hard condition, the nuclear shape factor was increased for the control condition, which resulted in a reduced ratio for treated vs. control cells on hard surfaces. Thus, we see again that a high cytokeratin-to-actin ratio was associated with more elongated cells on soft vs. stiff substrates, and treatment with TGF-β1 and hypoxia could be used to overcome this response to substrate stiffness.

### 3.6. Cytoskeletal Inhibitors Alter Morphological Response to TGF-β1 and Hypoxia Treatment

Next, we tested the effects of cytoskeletal inhibitors targeting Rho-associated Kinase (ROCK), myosin light chain kinase (MLCK), and the Rho/MRTF/SRF pathway on cell and nuclear shape and cytoskeletal protein expression. H1152 and ML7 were selective ROCK and MLCK inhibitors, respectively, and CCG1423 was a less selective Rho/MRTF/SRF pathway inhibitor. For these studies, TGF-β1 and hypoxia treatment were combined with DMSO vehicle control or ML7, H1152, or CCG1423 inhibitor treatments. The mean shape factors along with mean fluorescence intensities were tabulated for treated cells cultured on soft and hard substrates, with red indicating the highest and green the lowest values for all parameters (Table 2).

Cells treated with TGF-β1 and hypoxia were elongated for all conditions (CSF << 1), and H1152 treatment resulted in further elongation on both soft and hard substrates. TGF-β1- and hypoxia-treated cells also had slightly elongated nuclear structure (NSF < 1), with reduced NSF for cells cultured on soft vs. stiff substrates. The NSF increased with H1152 treatment, indicating that the nuclei were rounder. This also supported our previous hypothesis that subpopulations undergoing cell and nuclear elongation in response to TGF-B and hypoxia treatments were distinct from each other. H1152 treatment was also associated with reduced F-actin and reduced myosin expression and increased ratios of cytokeratin and myosin to F-actin; this cytoskeletal profile was similar for treated cells cultured on soft and hard substrates. The more elongated cell structure was likely correlated with the reduced cortical actin, whereas reduced myosin expression may lead to less tension on the nucleus and rounder nuclear structure.

With ML7 treatment, myosin expression was reduced, nuclear shape factors were increased, along with actin expression, which resulted in reduced cytokeratin-to-actin ratio. When these differences were normalized to DMSO treated control, it was clear that the effects of ML7 were less significant compared to H1152 and CCG1423 (Table 2B). The CCG1423 treatment had little effect on cell and nuclear shape; however, cytokeratin expression was increased, and actin expression was reduced for both substrate conditions, resulting in increased cytokeratin-to-actin and myosin-to-actin ratios. In summary, H1152 had the largest effect on cell and nuclear shape, which was correlated with a large reduction in F-actin expression; whereas CCG1423 had very little effect on cell and nuclear shape but resulted in the largest increase in cytokeratin expression. The CCG1423-treated cells also displayed more differences in their mechanical response to substrate stiffness with TGF-β1- and hypoxia-treated cells having lower actin and higher cytokeratin expression and increased myosin-to-actin ratio on soft relative to hard substrates.

## 4. Discussion

Extensive intratumor heterogeneity contributes to the lethality of epithelial ovarian cancers, which are a leading cause of cancer-related mortality in women [2,44,45,46]. The heterogeneity in EOC cells results from a combination of biophysical and biochemical cues that are present in the tumor microenvironment and can only be apparent by analyzing tumor cells at a single-cell or subpopulation level. Here, we investigated how chemical and physical aspects of the tumor microenvironment, including differences in matrix mechanics, growth factor signaling, and tissue hypoxia, influence heterogeneity in the degree of EMT in EOC cells. For these studies, EOC cells were cultured on 2D tissue culture plastic or polyacrylamide substrates with different rigidities, including soft substrates that mimic fatty tissues in the peritoneum and hard substrates that mimic dense tumors [47]. Heterogeneity in the EMT response was analyzed by tracking the morphological, cytoskeletal, and nuclear alterations in individual EOC cells. These studies demonstrate that EOC cells are spatially aware of their surroundings, with a subpopulation of EOC cells that are at the periphery of a clustered cells exhibiting a greater degree of EMT in response to TGF-β1 and hypoxia treatment than cells at the center of these epithelial cell clusters.

**Figure A5:**
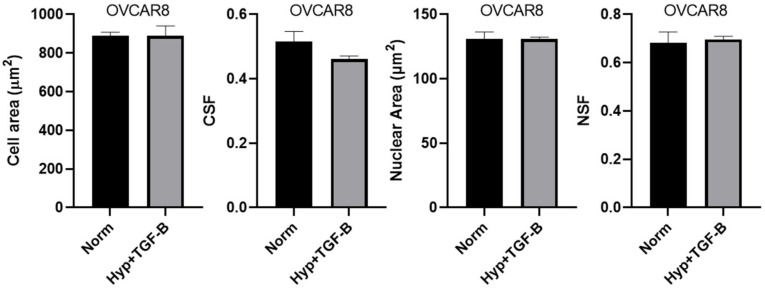
Morphological characterization of OVCAR8 cells cultured and treated on tissue culture plastic (TCP) with Hypoxia and TGF-β1 (50 ng/mL). Results are reported as average ± standard error of the mean.

Bulk measurements are often used to identify EMT; however, this approach often misses partial EMT, which is when epithelial cancer cells lose only some of their epithelial characteristics, demonstrating both mesenchymal and epithelial characteristics [48]. We found EMT gene expression analysis OVCAR3 to be confounding, as we found an increase in both epithelial and mesenchymal markers simultaneously (Figure A2A). We found even less response from OVCAR8 cells both genetically and morphologically after combination treatment (Figure A2B, Figure A5). It is likely that only a small subset of cancer cells exhibits partial EMT and are regulated by their environmental stimuli or proximity to other cells [48]. The extent of EMT was evaluated by observing cytoskeletal and nuclear shape changes as well as analyzing actomyosin and cytokeratin distribution in the OVCAR3 cells. Heterogeneity, defined by coefficient of variation (CV), was the largest in actin among the group of cytoskeletal proteins we probed (Table A4), followed by cytokeratin and myosin. We also observed higher CV on soft substrates post-treatment for myosin and cytokeratin driving cell heterogeneity. Finally, cells with elongated nucleus (NSF < 10th quantile) displayed more heterogeneous expression of all three cytoskeletal proteins than the elongated cells (CSF < 10th quantile) on soft substrates post treatment (Table A5). To analyze the role of actomyosin contractility, cells were treated with Rho-ROCK and MRTF-SRF pathway inhibitors. H1152 inhibited ROCK-mediated F-actin assembly and significantly lowered actin and p-Myo expression and their heterogeneity (Table A6). Interestingly, this resulted in an even more heterogeneous cell population with highly elongated cells. However, this treatment was successful in reversing TGF-β1- and hypoxia-induced nuclear elongation and heterogeneity.

Metastasizing cancer cells are required to squeeze both their cytoskeleton and nucleus for successful navigation and relocation to secondary sites. Morphologically, there are two types of serous ovarian cancer, low-grade type I and high-grade type II, with II being the more advanced, aggressive malignant form [49]. This is an important point of study, as there are multiple forms of ovarian cancer, all with varying degrees of invasiveness. Therefore, studying heterogeneity in ovarian cancer cells with varying degrees of an EMT phenotype is important to understanding the metastatic cascade and the development of new treatment strategies [50,51]. Our studies show that a small percentage of OVCAR3 cells demonstrate a significant induction of EMT and, therefore, a significant increase in metastatic potential.

**Figure A6:**
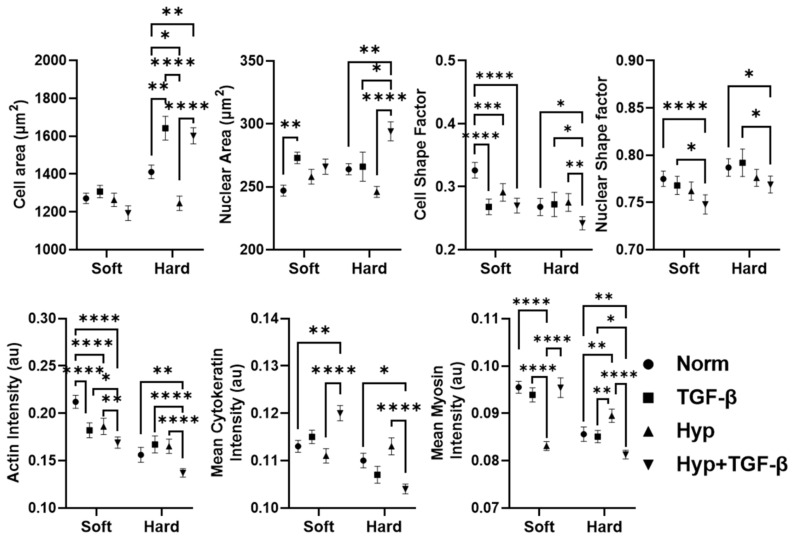
Comparison of the effects of individual TGF-B, and hypoxia treatment with their combined treatment on morphological properties and cytoskeletal organization of OVCAR3 cells cultured on polyacrylamide substrates using a two-way ANOVA analysis. Statistical significances are indicated as (*) for *p* ≤ 0.05, (**) for *p* ≤ 0.01, (***), *p* ≤ 0.001, and (****) for *p* ≤ 0.0001.

TGF-β and hypoxia are intricately linked with mechanosensing pathways and EMT. Both TGF-β and hypoxia can independently or synergistically activate EMT transcription factors including Twist, Snail, and Zeb-1 [52,53,54]. The effect of these biochemical cues on OVCAR3 cells individually were mostly attenuated on both rigidities (Figure A6), similar to the previously reported response of OVCAR3 cells to TGF-β treatment [55]. However, the combination of both resulted in additive or synergistic changes in cell and nuclear parameters as well as cytoskeletal protein expressions (Figure A6). Interestingly, hypoxia treatment elicited differential response of myosin and cytokeratin expressions compared to TGF-β treatment individually; for the combination treatment, these expressions followed the individual TGF-β treatment trend. These results suggested that hypoxia-driven additive or synergistic effects were likely TGF-β dependent and could differ significantly from the effects of hypoxia alone. The canonical TGF-β and hypoxia signaling enacted through SMADs, and hypoxia in-ducible factor (HIF) can significantly modulate EMT response of the OVCAR3 cells we have observed. Additionally, from ANOVA analysis, we found that treated cells showed significant interactions between substrate stiffness and the chemical cues, suggesting OVCAR3 cells becoming more aware of the substrate stiffness following treatment as they responded to these chemical cues (Table A7). Studying the spatial distribution of activated SMADs and HIFs in correlation to shape change could be pursued to gain further insight into the context-dependent activation EMT in epithelial ovarian cancer cells.

We previously showed that more metastatic ovarian cancer cells such as SKOV3 displayed a more invasive phenotype on soft vs. hard polyacrylamide substrates; however, in this study, less metastatic cell lines such as OVCAR3 displayed rigidity independent behavior [12]. These less invasive cells were also more epithelial and grew in epithelial cell clusters of varying sizes. Thus, we also analyzed the role of spatial heterogeneity in OVCAR3 cells to characterize the effects of these tumor microenvironment stimuli on peripheral and central cells within these epithelial clusters. Previous studies showed that cancer cells at the invading front were more likely to express mesenchymal markers associated with EMT—whereby less motile epithelial cancer cells transitioned into more invasive mesenchymal cells [22,24,56]. Cells at the invasive front were often more elongated with fewer cell–cell contacts; this was in contrast to cells at the core, which were more tightly packed in uniform circular shapes akin to a jammed state [22,57]. Enhanced proliferation of jammed tumor cells may trigger the unjamming of surrounding cells to induce this migratory behavior at the invading front. When densely packed cells migrated into tracks created by leader cells at the periphery, this unjamming effect may have promoted the collective invasion of the peripheral cells. As the combined treatment with TGF-β1 and hypoxia appeared to induce actomyosin reorganization to prepare cancer cells for migration—any peripheral EOC could potentially act as leader cell for collective cell migration but may need additional directional cues in the tumor microenvironment, such as platelet-derived growth factor (PDGF), for such changes to occur [58]. Thus, the spatial EMT response in EOC cells was likely driven by cellular location as opposed to intrinsic differences in cancer cells.

## 5. Conclusions

Taken together, these studies demonstrated that spatial organization and intracellular cytoskeletal properties of less invasive EOCs on 2D substrates played a profound role in determining varied EMT response. Moreover, it also demonstrated that the highly invasive properties of ovarian cancer were likely due to partial EMT of outlier peripheral populations. Other single cell analyses such as single-cell RNA-sequencing or fluorescence-activated cell sorting to identify EMT surface markers would also be useful in differentiating between these two populations. In conclusion, OVCAR3 cells treated with TGF-β1 and hypoxia cultured on substrates demonstrated that distinct subpopulations of the cells underwent either cell or nuclear elongation. This elongation effect was significantly more likely when those cells were cultured on softer substrates. This preference for soft substrates further highlighted the unusual behavior of ovarian cancer cells specifically, agreeing with the previous studies on stiffness-mediated malignancy [12]. This reflected the metastatic behavior of ovarian cancer, where cells disseminated into peritoneal fluid and adhered to softer tissues of the peritoneal cavity. This provided evidence for mechanosensitivity within the OVCAR3 cell line as a possible modulator of the response to TGF-β and hypoxia.

## Figures and Tables

**Table 1:** Cell and nuclear shape factors associated with the most extreme cytoskeletal structure differences. (**A**) To determine the effects of actin, cytokeratin, and p-Myo expression levels on cell and nuclear shape, we analyzed the single cell distribution of fluorescence intensities to identify cell populations with the highest (top 10%, hi) and lowest (bottom 10%, lo) levels of F-actin, cytokeratin, and p-Myo expression. The CSF and NSF values corresponding with low and high protein expressers are plotted along with high and low ratios of cytokeratin to F-actin and myosin to F-actin. The high and low expressers represent the cells in each population with the largest differences in cytoskeletal structure. (**B**) To determine if differences in cytoskeletal structure were correlated with the cell’s ability to respond to chemical and mechanical stimuli, we determined the ratio of cell and nuclear shape factors for TGF-β1- and hypoxia-treated cells relative to serum-free control cultured on soft and hard substrates. The heat map values highlight more elongated morphologies in green, rounder morphologies in red, and the statistically significant differences between treated and control cells are underlined for all *p*-values less than 0.05.

A								
	Cell Form Factor				Nuclear Form Factor			
	SF-Norm-Soft	TGF-Hyp-Soft	SF-Norm-Hard	TGF-Hyp-Hard	SF-Norm-Soft	TGF-Hyp-Soft	SF-Norm-Hard	TGF-Hyp-Hard
Actin-Lo	0.15	0.136	0.127	0.173	0.827	0.816	0.844	0.752
Actin-Hi	0.442	0.394	0.421	0.417	0.712	0.686	0.679	0.699
Myosin Lo	0.355	0.404	0.402	0.486	0.857	0.807	0.817	0.802
Myosin Hi	0.634	0.723	0.62	0.498	0.755	0.752	0.707	0.663
Cytokeratin-Lo	0.239	0.346	0.3	0.203	0.827	0.762	0.796	0.798
Cytokeratin-Hi	0.341	0.366	0.315	0.235	0.673	0.708	0.745	0.728
Cytokeratin to Actin-Lo	0.487	0.404	0.445	0.421	0.742	0.709	0.659	0.736
Cytokeratin to Actin-Hi	0.19	0.168	0.153	0.195	0.753	0.749	0.822	0.762
Myosin to Actin-Lo	0.489	0.436	0.444	0.43	0.694	0.672	0.662	0.697
Myosin to Actin-Hi	0.786	0.767	0.777	0.788	0.816	0.767	0.796	0.788
**B**								
**TGF-HYP/SF**								
	**Cell Form Factor**				**Nuclear Form Factor**			
	**Soft**		**Hard**		**Soft**		**Hard**	
Actin-Lo	0.906667		1.362205		0.986699		0.890995	
Actin-Hi	0.891403		0.990499		0.963483		1.029455	
Myosin Lo	1.138028		1.208955		0.941657		0.98164	
Myosin Hi	1.140379		0.803226		0.996026		0.937765	
Cytokeratin-Lo	1.447699		0.676667		0.921403		1.002513	
Cytokeratin-Hi	1.073314		0.746032		1.052006		0.977181	
Cytokeratin to Actin-Lo	0.829569		0.946067		0.955526		1.116844	
Cytokeratin to Actin-Hi	0.884211		1.27451		0.994688		0.927007	
Myosin to Actin-Lo	0.891616		0.968468		0.9683		1.05287	
Myosin to Actin-Hi	0.975827		1.014157		0.939951		0.98995	

**Table 2:** Combined treatment with TGF-β1 and hypoxia and cytoskeletal inhibitors alters cell and nuclear shape and cytoskeletal protein expression. (**A**) TGF-β1 and hypoxia treatment were combined with DMSO vehicle control or ML7 (targeting MLCK), H1152 (targeting ROCK), or CCG1423 (targeting Rho/MRTF/SRF pathway) cytoskeletal inhibitor treatments. Heat map of the cell and nuclear shape factors, mean fluorescence intensities for F-actin, cytokeratin, and p-Myo, and ratios of protein expression, with red indicating the highest value and green the lowest for all parameters. These data show ROCK inhibition has the largest effect on cell and nuclear shape; whereas, the Rho/MRTF/SRF inhibitor has little effect on cell and nuclear shape but increases cytokeratin expression. (**B**) Cytoskeletal inhibitor results were normalized to DMSO condition to look at the direct results of cytoskeletal inhibitors on the TGF-β1 and hypoxia treatment conditions on soft and hard substrates. These data show that ML7 treatment results in less significant differences in comparison to H1152 and CCG1423 treatments. Statistically significant differences between inhibitor-treated and DMSO-treated cells are underlined for all *p*-values less than 0.05.

**A**								
	**TGF-Hyp-Soft**				**TGF-Hyp-Hard**			
	**DMSO**	**ML7**	**H1152**	**CCG1423**	**DMSO**	**ML7**	**H1152**	**CCG1423**
Cell form factor	0.298	0.306	0.217	0.324	0.334	0.329	0.234	0.332
Nuclear form factor	0.617	0.66	0.748	0.652	0.677	0.714	0.772	0.7
Mean Actin	0.148	0.134	0.099	0.117	0.133	0.132	0.097	0.123
Mean Cytokeratin	0.132	0.116	0.122	0.167	0.131	0.123	0.12	0.156
Mean Myosin	0.074	0.07	0.067	0.073	0.073	0.069	0.068	0.073
Cytokeratin to Actin	1	0.888	1.247	1.341	1.012	0.941	1.261	1.361
Myosin to Actin	0.502	0.541	0.688	0.715	0.572	0.58	0.712	0.598
**B**								
	**TGF-Hyp-Soft (Normalized to DMSO)**				**TGF-Hyp-Hard (Normalized to DMSO)**			
	ML7	H1152	CCG1423		ML7	H1152	CCG1423	
Cell form factor	1.027	0.728	1.087		0.985	0.701	0.994	
Nuclear form factor	1.070	1.212	1.057		1.055	1.140	1.034	
Mean Actin	0.905	0.669	0.791		0.992	0.729	0.925	
Mean Cytokeratin	0.879	0.924	1.265		0.939	0.916	1.191	
Mean Myosin	0.946	0.905	0.986		0.945	0.932	1.000	
Cytokeratin to Actin	0.888	1.247	1.341		0.930	1.246	1.345	
Myosin to Actin	1.078	1.371	1.424		1.014	1.245	1.045	

## Data Availability

The data that support the findings of this study are available from the corresponding author M.R.D. upon reasonable request.

## Appendix A. Methods

### Appendix A.1. Cell Culture

Ovarian carcinoma cell line OVCAR3 was purchased from ATCC and cultured per manufacturer’s instruction. Another ovarian cancer cell line OVCAR8 was provided generously by Dr. Richard Freiman. Cells were cultured in RPMI media supplemented with 10% FBS and 1% penicillin streptomycin. All cells were incubated at 5% CO_2_ and 37 °C. The cells were treated with TGF-β at 50 ng/mL and were allowed to incubate for 48 h before further analysis or downstream experiments. Cells treated with hypoxia were cultured in 2.5% O_2_.

### Appendix A.2. Mechanical Testing of Polyacrylamide Gels

In order to determine mechanical properties of the polyacrylamide (PA) substrates, samples were tested using the Electroforce 3100 (Texas Instruments, Dallas, TX, USA). To control the size and shape of the PA gels, soft and hard substrates with previously described formulations (Table A1) were added to a 10 mL test tube containing polymerized 1% agarose at the bottom of the tube. The surface area and length of the PA samples were measured before testing. Then, the bulk mechanical properties of the gel were evaluated with three samples mechanically tested for each of the soft and hard substrates. The Young’s Modulus (E) was calculated from the slope of the linear region in the force versus displacement curve, where E = (F/A)/(∆L/L) = (Stress/Strain). The experimentally calculated average kilopascal values for both soft and hard substrates can be found in Table A1.

**Table A1:** Polyacrylamide Substrate Concentrations. The table shows the final concentration of acrylamide and bis-acrylamide in soft and hard substrates.

Young’s Modulus	Final Acrylamide Concentration	Final Bis-Acrylamide Concentration
2.6 kPa (Soft)	10%	0.03%
36.2 kPa (Hard)	10%	0.30%

### Appendix A.3. RNA Isolation and Quantitative Real-Time Polymerase Chain Reaction (qRT-PCR)

RNA was isolated from the OVCAR3and OVCAR8 cells using the TRIzol reagent (Thermo Fisher Scientific, Waltham, MA, USA) according to the manufacturer’s protocol. After confirming the purity of the isolated RNA, using Nanodrop 1, cDNA was synthesized by reverse transcription using the BIO-RAD iScript cDNA synthesis kit. To evaluate the effect of TGF-β and hypoxia on the OVCAR cell lines, we ran a panel of primer consisting of both epithelial and mesenchymal markers (Table A2). Expression of all genes quantified was normalized to expression of 18s RNA for an endogenous control. A list of primers used can be found in Table A2.

**Table A2:** Primers Used for Epithelial and Mesenchymal Markers. The table shows all primers used in qRT-PCR for epithelial and mesenchymal markers in addition to the endogenous control.

	Marker	Forward Sequence	Reverse Sequence
Epithelial Markers	E-Cadherin (*ecad*)	CTCGTAACGACGTTGCACC	CTGTGGGGTCAGTATCAGCC
	Cytokeratin 14 (*krt14*)	GGAGGAGATGAATGCCCTGA	GTGAAGAACCATTCCTCGGC
	Cytokeratin 19 (*krt19*)	CGCGACTACAGCCACTACTA	AGAGCCTGTTCCGTCTCAAA
Mesenchymal Markers	N-Cadherin (*ncad*)	CCATCAAGCCTGTGGGAATC	CTGTGGGGTCATTGTCAGCC
	Snail (*snail*)	ACCACTATGCCGCGCTCTT	GGTCGTAGGGCTGCTGGAA
	Vimentin (*vim*)	CTGGATTCACTCCCTCTGGTTG	GGTCATCGTGATGCTGAGAAG
	ZEB1 (*zeb1*)	TTCAAACCCATAGTGGTTGCT	TGGGAGATACCAAACCAACTG
Control	18s rRNA	GTAACCCGTTGAACCCCATT	CCATCCAATCGGTAGTAGCG

### Appendix A.4. Semi-Automated Analysis of OVCAR3 Cell Images with CellProfiler

High magnification images in 2D were analyzed using custom-built pipelines in CellProfiler. Segmented images were first saved and manually sorted based on the accuracy of drawn cell boundaries in CellProfiler (Figure A1A). Images with good segmentation were subsequently analyzed for morphological and cytoskeletal protein characterization. Furthermore, we quantified normalized radial distribution of all the probed cytoskeletal proteins in the cells with low and high expression (Figure A1B).

## Appendix B. Data

**Table A3:** Quantification of nuclear and cellular morphological heterogeneity using coefficient of variation (CV) analysis. CV_10 and CV_50 quantify variation in shape parameters in the elongated subpopulation with shape factor below 10th and 50th quantile, whereas CV_All depicts the variance in the whole population.

	SF-Norm-Soft	TGF-Hyp-Soft	SF-Norm-Hard	TGF-Hyp-Hard
NSF				
CV_All	0.15	0.18	0.14	0.16
CV_50	0.17	0.20	0.16	0.19
CV_10	0.16	0.25	0.11	0.31
**CSF**				
CV_All	0.56	0.59	0.60	0.61
CV_50	0.33	0.37	0.36	0.35
CV_10	0.34	0.28	0.29	0.29

**Table A4:** Mean and coefficient of variation (CV) of cytoskeletal protein expression.

	SF-Norm-Soft		TGF-Hyp-Soft		SF-Norm-Hard		TGF-Hyp-Hard	
	Mean	CV	Mean	CV	Mean	CV	Mean	CV
Mean Actin	0.212	47.12	0.169	47.46	0.156	59.84	0.137	45.44
Mean Myosin	0.095	16.82	0.095	23.56	0.086	16.04	0.081	10.62
Mean Cytokeratin	0.113	28.73	0.120	32.03	0.110	30.07	0.104	23.71

**Table A5:** The coefficient of variation (CV) of cytoskeletal parameters in cells with elongated cell body (CSF < 10th quantile) and elongated nuclei (NSF < 10th quantile).

CV	Cell Shape Factor < 10th Quantile				Nuclear Shape Factor < 10th Quantile			
	SF-Norm-Soft	TGF-Hyp-Soft	SF-Norm-Hard	TGF-Hyp-Hard	SF-Norm-Soft	TGF-Hyp-Soft	SF-Norm-Hard	TGF-Hyp-Hard
Mean Actin	32.85	24.32	15.00	28.10	41.20	41.44	52.80	46.18
Mean Myosin	16.26	9.97	13.67	12.61	15.19	19.15	14.48	10.75
Mean Cytokeratin	31.11	33.32	37.14	16.37	25.85	39.50	39.54	22.68
Cytokeratin to Actin	26.58	28.37	29.67	40.93	58.23	74.1	61.85	42.65
Myosin to Actin	28.49	19.04	29.18	16.95	67.4	45.51	62.38	31.78

**Table A6:** Coefficient variation (CV) of morphological and cytoskeletal parameters after inhibitor treatment (H1152).

CV	TGF-Hyp-Soft		TGF-Hyp-Hard	
	DMSO	H1152	DMSO	H1152
Cell shape factor	54.08	68.63	56.60	75.70
Nuclear shape factor	24.87	17.64	20.92	14.98
Mean Actin	30.02	17.11	22.39	18.90
Mean Cytokeratin	33.19	30.39	33.13	31.18
Mean Myosin	9.60	4.06	7.314	5.00
Cytokeratin to Actin	28.73	28.20	32.75	32.15
Myosin to Actin	21.89	11.72	17.98	12.84

**Table A7:** Comparison of ANOVA analysis results of morphological properties and cytoskeletal organization results using two- vs. four-treatment groups. Statistical significances are indicated as (*) for *p* ≤ 0.05, (**) for *p* ≤ 0.01, (***), *p* ≤ 0.001, and (****) for *p* ≤ 0.0001.

Two-Way Analysis with Four Groups	Interaction	Row Factor (Substrate Stiffness)	Column Factor (SF/TGF-β ± Hyp)
NSF	0.62 (ns)	<0.0001 (****)	<0.0001 (****)
Nuclear Area	0.0016 (**)	0.1393 (ns)	<0.0001 (****)
CSF	0.0002 (***)	<0.0001 (****)	<0.0001 (****)
Cell Area	<0.0001 (****)	<0.0001 (****)	<0.0001 (****)
Actin	<0.0001 (****)	<0.0001(****)	<0.0001 (****)
Cytokeratin	<0.0001 (****)	<0.0001 (****)	0.9187 (ns)
Myosin	<0.0001 (****)	<0.0001 (****)	<0.0001 (****)
**Two-way Analysis with Two Groups**	**Interaction**	**Row Factor** **(Substrate Stiffness)**	**Column Factor** **(SF/TGF-** **β + Hyp)**
NSF	0.3396 (ns)	<0.0005 (***)	<0.0001 (****)
Nuclear Area	0.3520 (ns)	<0.0001 (****)	<0.0001 (****)
CSF	0.0162 (*)	<0.0001 (****)	<0.0001 (****)
Cell Area	<0.0003 (***)	<0.0001 (****)	0.1270 (ns)
Actin	<0.0002 (***)	<0.0001 (****)	<0.0001 (****)
Cytokeratin	<0.0001 (****)	<0.0001(****)	0.7150 (ns)
Myosin	<0.0111 (*)	<0.0001 (****)	<0.0078 (**)
